# Supplementary material for: Signal-to-Noise Analysis Can Inform the Likelihood That Incidentally Identified Variants in Sarcomeric Genes Are Associated with Pediatric Cardiomyopathy
Source: J Pers Med. 2022 Apr 30;12(5):733. doi: 10.3390/jpm12050733 (PMC9145017; doi:10.3390/jpm12050733)
Supplement: Supplementary file 1 [file jpm-12-00733-s001.zip › HCM ES Variants Manuscript, Supplemental .pdf]

SUPPLEMENTAL MATERIALS

Signal-to-noise analysis can inform the likelihood that incidentally identified variants in sarcomeric genes are associated with pediatric cardiomyopathy

Leonie M. Kurzlechner BS, Edward G. Jones MD, Amy M. Berkman MD, Hanna J. Tadros MBBCh, Jill A. Rosenfeld MS, Yaping Yang PhD, Hari Tunuguntla MD, Hugh D. Allen MD, Jeffrey J. Kim MD, Andrew P. Landstrom MD, PhD

SUPPLEMENTAL METHODS:

Study Cohorts

ES Cohort

This research study was approved by the Baylor College of Medicine and Duke University Health System Institutional Review Boards, respectively (IRB Pro00094341). The ES cohort has been previously described.[1] Briefly, this cohort was comprised of individuals referred for clinical ES to the Baylor Genetics Laboratories (Houston, Texas, United States) independent of referral diagnosis or indication for genetic testing. Individuals included in this cohort were genetic testing probands. Roughly 90% were referred from institutions within the United States and ~47% within the state of Texas. While not exclusively pediatric, most underwent genetic testing during childhood. Individuals excluded from this cohort were 1) non-proband family members and 2) those whose samples were derived for platform validation studies or from oncological samples. Trio data for probands in the ES cohort was manually curated. Variants were derived from the report provided to the referring physician as well as the "expanded" report of variants only reported on request; variants on both reports were included to remove any potential practitioner bias. Variants included in this cohort were: 1) identified in the coding nucleotide sequence or predicted splice junction of an hypertrophic cardiomyopathy (HCM)-associated gene locus, 2) potential splice donor or splice acceptor-effecting variants located within the first four nucleotides near the splice junction, and 3) deemed "pathogenic", "likely pathogenic", or "variant of uncertain significance" (VUS) at the time of genetic testing according to American College of Medical Genetics (ACMG) interpretation guidelines. Variants excluded from this cohort were 1) interpreted as "benign" or "likely benign" at the time of genetic testing, 2) intronic variants outside of the first four nucleotides of the splice junction, 3) 5'or 3' untranslated region variants, or 4) synonymous variants. To account for recent changes in research-based evidence of pathogenicity since the initial identification of the variant at time of genetic testing, each variant was further verified for pathogenicity via ClinVar aggregate records.[2] All variants holding a clinical significance of "benign" or "likely benign" were excluded.

HCM Case Cohort

The HCM case cohort was based on multiple cohort-based studies abstracted from the literature (**Supplemental Table S1**). Variants were included that were deemed LP/P or VUS and were identified in patients who met clinical criteria for HCM. Similar to the ES cohort, to account for changes in evidence of pathogenicity since publication, all variants holding a clinical significance of "benign" or "likely benign" in ClinVar aggregate records were excluded. This cohort consisted of 6,995 individuals across 15 independent studies, with cross-sectional or prospective design, that performed comprehensive genetic sequencing on individuals who met clinical diagnostic criteria for HCM.[3-17]

Control Cohort

The control cohort was comprised of rare genetic variants derived from the Genome Aggregate Database (gnomAD) which consisted of individuals of African/African American (N=12,013), Ashkenazi Jewish (N=5,076), East Asian (N=9,435), Finnish (N=12,897), Non-Finnish European (N=63,352), Latino (N=17,210), South Asian (N=15,391), and other racial individuals (N=3,232) for a total of 138,632 individuals.[18] While the gnomAD database is comprised partly of various disease-specific cohorts, including cardiac disease and population genetics studies, it excludes individuals known to have severe pediatric disease as well as severe disease in their first-degree relatives; therefore, we utilized these individuals as "control" or "reference" alleles. Given the observation of likely pathogenic variants found among gnomAD cases, we determined the maximum credible minor allele frequency (MAF) of gnomAD-identified pathogenic variants and set an inclusion threshold of MAF <0.0001 such that all likely pathogenic variants would fall below this threshold. This MAF has been previously utilized in the setting of cardiomyopathies.[19-21] The inclusion and exclusion criteria detailed for the ES cohort were also applied to the gnomAD cohort, with the exception of interpretations of variant pathogenicity, which are not available for these control data. Given limitations of the gnomAD variant reporting, and the inability to identify multiple variant-positive individuals, MAF was used to determine prevalence. Overall rare variant frequency was calculated by combining variant-specific MAF.

TCH Cohort

The Texas Children's Hospital (TCH) cohort was comprised of patients within the clinical ES cohort who hosted a rare sarcomeric variant in an HCM-associated gene who were referred from Texas Children's Hospital, Baylor College of Medicine (Houston, Texas, United States). These patients were identified based on referral records and cross-referencing with clinical encounters. Anonymous clinical information including basic demographics, clinical evaluation and diagnoses, and transthoracic echocardiographic findings were reviewed retrospectively. Patients referred for evaluation by pediatric cardiology were noted and their records were reviewed for clinical suspicion, or diagnosis, of HCM. Patients with intraventricular septal thickness >2 standard-deviations from body surface area-normalized size (Z-score) without a subsequent normal measurement or a clinical suspicion for HCM noted on their clinical evaluation had their echocardiogram and diagnoses reviewed by a pediatric cardiologist with expertise in diagnosis of cardiomyopathies. Patients were excluded who did not have an echocardiogram, had hemodynamically significant structural abnormality, or had evidence of hypertrophy that resolved on subsequent echocardiograms. Post-cardiac transplant echocardiograms were not included in this analysis. As the TCH cohort was a subset of the ES cohort, patients from this cohort are included as probands in the ES cohort.

Signal-to-noise analysis

Topology and variant mapping were conducted utilizing consensus primary sequences from Ensembl browser.[22] The frequency of an ES or HCM cohort variant at a given amino acid position was determined as previously described.[23, 24] S:N plots compared variant frequency in coding regions of HCM-related genes (*MYBPC3, MYH7, TNNI3, TPM1, TNNT2, MYL2, MYL3, ACTC1, TNNC1*) between ES and HCM cohorts to rare (MAF < 0.0001) variants in the gnomAD population database. Weighted variant frequencies and S:N cut-offs to determine whether incidental variant frequencies were statistically different from known HCM-associated variant frequencies were calculated, as previously described.[25] After establishing these gene-specific S:N cutoffs, we stratified our analysis by variant type (missense versus radical) and determined type-specific S:N cutoffs.

We used S:N to determine mutation “hot spots” which were defined as areas of the primary amino acid sequence exceeding the applicable S:N threshold. We then applied this to current ACMG criteria and performed variant re-classification. During reclassification, we applied PM1 criteria to variants with S:N above the gene-specific threshold. As part of variant interpretation using ACMG guidelines, we performed a comprehensive search of ClinVar to assess ES variants for any with the same amino acid change as known pathogenic variants (PS1 criteria), to evaluate any functional evidence of a damaging gene effect (PS3), to identify other pathogenic missense variants at the same amino acid position (PM5), and to determine whether the variant had at least one recent classification as pathogenic (PP5).

Statistics

Statistical results were expressed as mean with variance expressed as standard deviation or median and interquartile range (brackets), as appropriate. Variance of prevalence/proportion was expressed as the exact 95% confidence interval (brackets). Comparisons were made by Student’s t-Test, Fisher’s Exact test, Chi-Square with Yates Correction, Sign test, and test of multiple proportions as appropriate using OpenEpi.[26] Gene-specific S:N thresholds were set using the Shapiro-Wilk test, Wilcoxon rank sum test, and Cohen’s d analysis with significance level set at 0.05 and power at 90%.[25] Software used included R version 3.4.1 (R core team, Vienna, Austria), SPSS Version 25 (SAS Institute, Cary, NC, USA), and GraphPad Prism version 9.1.2 (GraphPad Software, San Diego, CA, USA). Statistical significance threshold was set at $P<0.05$.

SUPPLEMENTAL RESULTS

Frequency of ES variants compared to control and HCM-afflicted individuals

To explore this disparity among the global frequency of each cohort, we stratified the analysis by mutation class. Among individuals undergoing ES testing, 482 probands (7.1% [6.5-7.8]) hosted a missense variant while 37 (0.5% [0.4-0.7]) hosted a radical variant; 10 probands hosted both a missense and a radical variant (0.1% [0.1-0.2]). The variants found in the ES cohort are listed in **Supplemental Table S4**. This strong predominance of missense variant-positive individuals was mirrored in the gnomAD cohort, which had a missense and radical variant-positive individual frequency of 4.7% [4.6-4.8] and 0.6% [0.5-0.6], respectively. The higher overall frequency of HCM case-associated variants was driven by a higher frequency of missense-positive individuals, with 2,004 individuals (32.3% [31.2-33.5]) hosting a missense variant, and by a nearly 20-fold increase in the frequency of radical variants compared to both ES and controls, with 716 (11.0% [10.3-11.8]) radical variant-positive cases. The similarity between the ES and control cohorts was highlighted in the relative proportion of total variants that were missense and radical (93.2% vs. 89.2% missense and 6.8% vs. 10.0% radical, respectively). In comparison, the HCM cohort relative frequency for missense and radical variants was 73.7% and 26.3%, respectively. These results are summarized in **Figure 2**.

Gene and variant type-specific ES variant distribution compared to control and HCM-afflicted individuals

To determine the prevalence of radical and missense variants in HCM-associated genes among the ES cohort, subset analysis of the variants based on variant type was conducted. Variants in the ES cohort mostly localized to *MYBPC3* (3.2% of the cohort) and *MYH7* (2.1%). The gnomAD cohort demonstrated a similar prevalence among genes to the ES cohort, with 1.6% of the cohort hosting variants in each *MYBPC3* and *MYH7*. In contrast, the HCM cohort demonstrated a disproportionately higher yield of variants compared with the other cohorts, though variants were similarly localized predominantly to *MYBPC3* (20.6% of the cohort) and *MYH7* (14.7%).

Among all ES variants, 504 (93.2%) were missense variants and 37 (6.8%) were radical variants; among unique variants, 371 (93.0%) were missense variants and 28 (7.0%) were radical variants. Most ES missense variants were found in *MYBPC3* (3.1% of the cohort) and *MYH7* (2.0%). This was mirrored in the gnomAD cohort, with 1.5% of the cohort hosting missense variants in both *MYBPC3* and *MYH7*. The HCM case cohort displayed a much higher frequency of missense variants in both *MYBPC3* and *MYH7*, with 10.3% and 13.5%, respectively. Radical variants were less common overall and were identified in the ES cohort most commonly in *TPM1* (0.16%) and *MYBPC3* (0.13%). The gnomAD control had a comparable distribution of radical variants, with *TPM1* (0.25%) and *MYBPC3* (0.11%) being the most common. In the HCM cohort, radical variants were enriched nearly 80-fold in *MYBPC3*, with 9.4% of the HCM cohort hosting a radical variant in that gene. This indicates that *MYBPC3* radical variants are overrepresented in the HCM cohort when compared to the ES cohort and gnomAD controls. In contrast, *MYBPC3* variants in the gnomAD and ES cohorts were mostly missense, with only 0.1% of each the gnomAD and ES cohorts found to have radical *MYBPC3* variants. Following *MYBPC3*, radical variants in the HCM cohort were most frequent in *MYH7* (0.37%) and in *TNNT2* (0.37%). These results are summarized in **Supplemental Figure S1**.

Gene-level signal-to-noise calculations between ES and pathogenic HCM variants

Given the relatively high degree of background genetic variation in HCM-associated genes among control individuals, we next calculated a gene-specific S:N by normalizing the frequency of variants in HCM and ES cohorts, respectively, against the background rate of variants in gnomAD controls. Across all nine genes, the global S:N ratio among HCM cases was 6.0. When applied to HCM cases, we identified several disease-associated genes with ratios greater than 6.0. Of the nine sarcomeric genes analyzed, *MYBPC3* demonstrated the highest S:N ratio (12.6 [12.5-12.6]), which was likely driven by a much higher overall *MYBPC3* variant prevalence in the HCM cohort (20.6%) relative to the gnomAD cohort (1.6%). Four other genes also generated high S:N ratios greater than 6.0 among individuals with a diagnosis of HCM, including *MYH7* (9.2 [9.2-9.3]), *TNNI3* (8.6 [8.4-8.8]), *TNNT2* (7.4 [7.2-7.6]), and *MYL2* (6.4 [6.2-6.7]). *ACTC1* also

demonstrated a high S:N ratio of 4.8 [4.4-5.3], though it was less than 6.0. *MYL3* yielded a borderline S:N ratio of 2.4 [2.1-2.8]. The remaining two genes had S:N ratios that were not significantly above 1.0 (*TPM1* (1.1 [0.9-1.3]) and *TNNC1* (1.1 [0.2-2.0])). These results are summarized in **Figure 3A**.

With the high S:N ratio among pathogenic variants in well-established sarcomeric HCM-associated genes noted in our HCM cohort, we next examined the S:N ratio for ES variants relative to the gnomAD control cohort. Among ES referrals, seven of the HCM-associated genes had borderline ratios: *MYL2* (2.3 [1.9-2.6]), *MYBPC3* (2.0 [1.8-2.1]), *TNNT2* (2.0 [1.6-2.3]), *MYH7* (1.3 [1.1-1.5]). The S:N ratios for the remaining genes (*MYL3*, *ACTC1*, *TPM1*, *TNNI3*, *TNNC1*) did not significantly exceed 1.0. In all genes aside from *TNNC1* and *TPM1*, the pathologic S:N ratio for the HCM cohort was significantly higher than the ratio found in ES referrals ($P<0.05$). These results are summarized in **Figure 3B**. Overall, the significant differences from gene to gene in HCM:gnomAD signal-to-noise suggest that variants identified in certain genes with higher signal-to-noise ratios should carry a higher diagnostic weight.

<u>Amino acid-level signal-to-noise stratified by missense versus radical variants</u>

After refining our signal-to-noise analysis to the amino acid level to inform the ACMG PM1 criteria, we further stratified our analysis to differentiate between signal generated by missense versus radical variants to address potential differences in pathogenicity of these variant types. To do this, we generated missense and radical signal-to-noise graphs that plotted S:N ratios either only for missense variants or only for radical variants, respectively. In *MYBPC3* there was overlap between the HCM:gnomAD signal generated by missense versus radical variants (**Supplemental Figure S2**), while in *MYH7* there is a far greater contribution of missense variants to pathogenic hotspots (**Supplemental Figure S3**). The S:N analysis of minor genes is detailed in **Supplemental Figures S4-10**. In addition to calculating global thresholds for meeting PM1 criteria for each gene, we also set gene-specific thresholds for missense and radical variants. Given that only *MYBPC3*, *MYH7* and *TPM1* yielded significant numbers of radical variants, we were not able to calculate S:N cutoffs for radical variants in the remaining genes. Amino acid locations where either

the global, missense, or radical HCM:gnomAD signal-to-noise ratio exceeds the respective threshold are listed in **Supplemental Table S5**.

Amino acid-level signal-to-noise ratio of domain-specific genetic variability in minor HCM-associated genes

To further characterize domain-specific S:N ratios, genes rarely associated with the pathogenesis of HCM were interrogated. Given the lower prevalence of variants found in phenotype-positive individuals of the HCM cohort in other genes, most did not reveal any significant domain-specific trends in signal-to-noise mapping. However, *TPM1* did have elevated S:N ratios that tracked to period 5, a region shown to be critical for TPM1-actin interactions.[27] These results are summarized in **Supplemental Figure S7**.

Pre-test clinical suspicion of HCM among ES referrals

To determine whether individuals referred for ES genetic testing demonstrated pre-ES suspicion for HCM, the clinical indications for ES referral were compiled. Of the 509 variant-positive probands in the ES cohort, the indication for genetic testing was concern for cardiovascular disease in a minority of patients. Of this cohort, 79.3% were referred for ES due to neurologic concerns; most common among these concerns were developmental delay (58.7%), abnormal tone (47.3%), seizures (30.6%), and autism (12.7%). Cardiac disease was included as part of the clinical phenotype indicated in the referral in 20.6% of the cohort. Only 14 variant-positive individuals (2.6%) in the cohort were referred solely for cardiovascular issues, of which 11 (2.1%) were for concern for cardiomyopathy, and six (0.7%) for HCM specifically. Overall, these findings suggest a low pre-test clinical suspicion of HCM among clinical ES referrals.

SUPPLEMENTAL TABLES

Supplemental Table S1. ES cohort variants showing evolving classification from time of testing to recent re-evaluation.

Separate Excel File

AA, amino acid; F, female; Het, heterozygous; Hom, homozygous; M, male; VUS, variant of uncertain significance

Supplemental Table S2. Summary of cardiomyopathy cohort variants

Total Cohort Size	Genotype-positive Individuals	*MYH7*	*MYBPC3*	*MYL2*	*MYL3*	*ACTC1*	*TPM1*	*TNNT2*	*TNNI3*	*TNNC1*	Reference
2912	917	302	465	21	6	4	24	33	43	0	Alfares et al.
90	32	13	10	3	1	1	1	2	3	0	Anerson et al.
722	207	65	123	11	1	NA	NA	5	8	NA	Berge et al.
1053	359	121	182	9	1	2	6	3	13	4	Bos et al.*
77	41	9	27	0	NA	NA	NA	5	2	NA	Brito et al.
108	36	14	20	NA	NA	NA	1	1	1	0	Erdmann et al.
471	163	64	70	1	3	2	3	11	4	NA	Gruner et al*
192	92	29	54	NA	NA	NA	NA	9	6	NA	Millat et al.
46	13	2	10	1	0	0	0	0	1	NA	Morner et al.
203	126	39	73	7	NA	1	2	8	2	NA	Olivotto et al.
197	124	50	52	5	1	NA	NA	8	8	NA	Richard et al.
130	33	13	20	NA	NA	NA	NA	NA	NA	NA	Rodriguez-Garcia et al.
236	97	39	60	NA	NA	NA	NA	NA	NA	NA	Waldmuller et al.
358	159	53	75	NA	NA	NA	NA	NA	NA	NA	Weissler-Snir et al.*
200	102	52	36	2	3	3	3	8	7	NA	Zou et al.

* Genotype data not available for individuals with >1 mutation

Supplemental Table S3. Sign test comparison of gene-specific frequencies

Parameter	Pairs	Median [CI]	P-value
Prevalence of (gnomAD – ES)	9	-0.09 [-0.46, 0.09]	0.18
Prevalence of (gnomAD - HCM)	9	-1.07 [-12.27, -0.10]	<0.01
Prevalence of (ES - HCM)	9	-0.82 [-11.82, -0.23]	<0.05

ES, exome sequencing; HCM, hypertrophic cardiomyopathy

Supplemental Table S4. Amino acid positions with signal-to-noise ratio exceeding gene-specific threshold

Separate Excel File

S:N, signal-to-noise, X denotes position is above threshold in respective column header

Supplemental Table S5. TCH cohort demographics

Characteristic	N (%)
Variant-Positive Probands	171
Total Families	171
Male	96 (56.1%)
Female	75 (43.9%)
Fetal	0
Age at Genetic Testing (y)	6.8 [1d – 52y]
Ethnicity	
East Asian	3.5%
African	12.3%
Caucasian	38.6%
GME	3.5%
Hispanic	41.5%
Pacific Islander	0.6%
Unique Variants	148
LP/P	7 (4.7% [1.3-8.2])
VUS	141 (95.3% [91.9-98.7])
Probands with 1 Variant	162 (94.7% [91.4-98.1])
Probands with 2 Variants	9 (5.3% [1.9-8.6])

LP/P, Likely Pathogenic/Pathogenic; VUS, Variant of Uncertain Significance; GME, Greater Middle East

SUPPLEMENTAL FIGURES

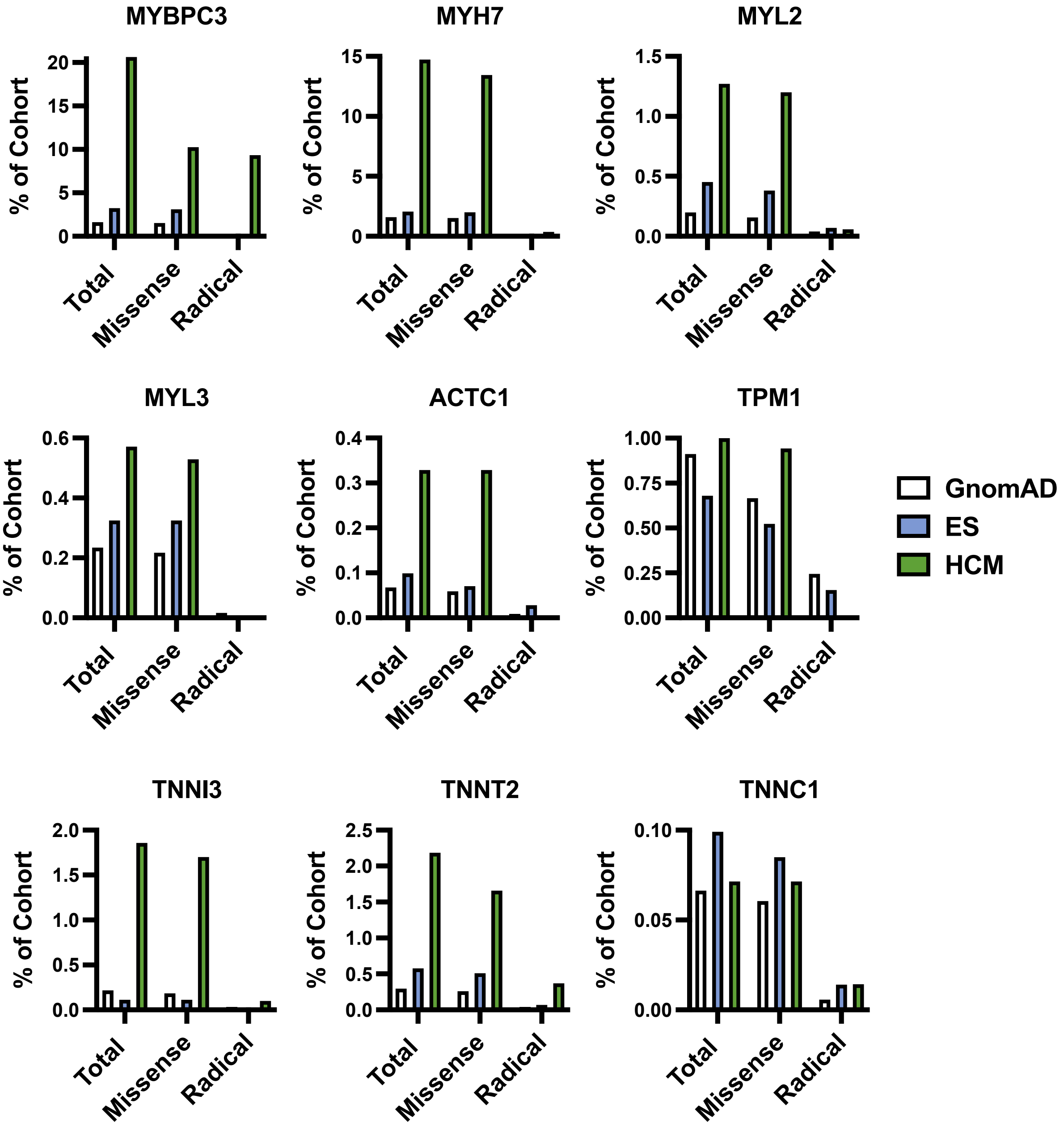

<u>Supplemental Figure S1</u>: Bar graphs depicting the distribution of total, missense, and radical variants across the gnomAD (white), ES (blue), and HCM (green) cohorts for each gene.

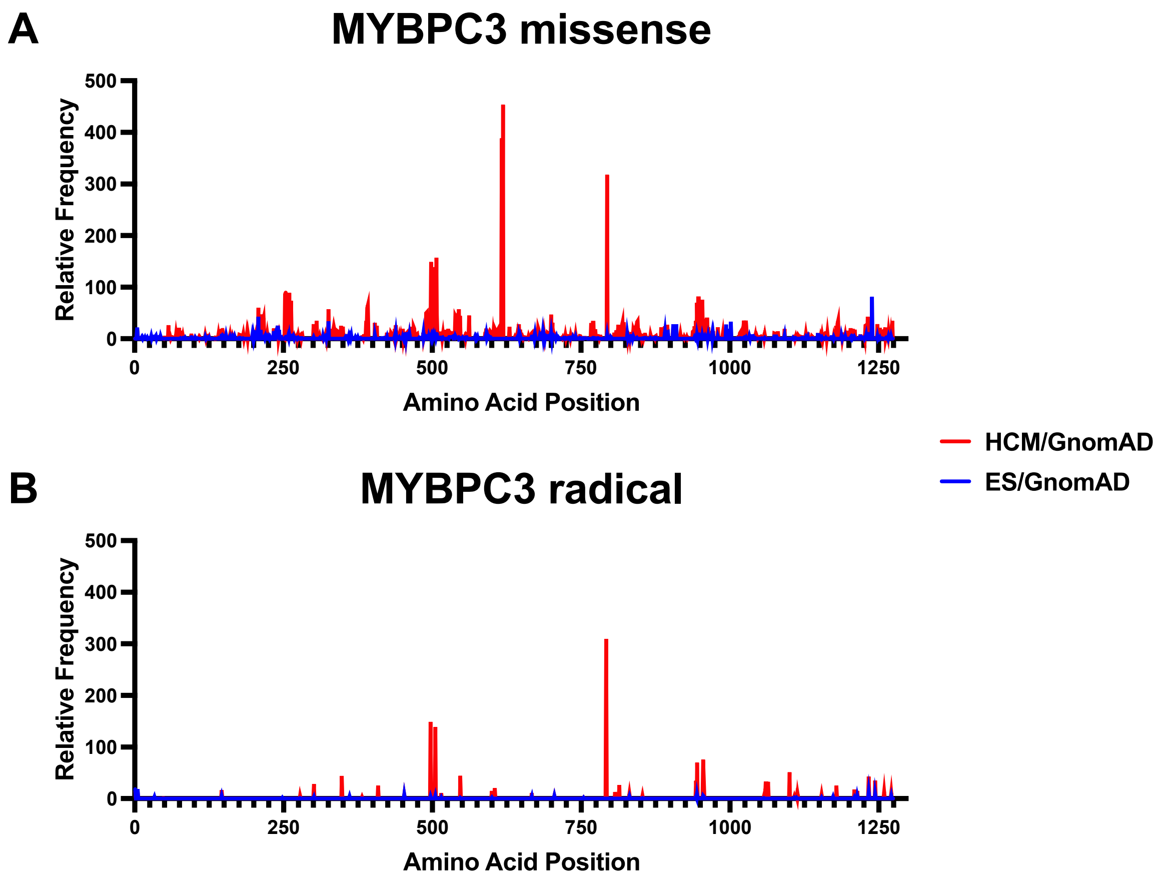

<u>Supplemental Figure S2:</u> A, Amino acid-level signal-to-noise analysis of missense variants found in *MYBPC3* for both HCM cases (red) and ES-identified variants (blue), compared with variants found in the gnomAD cohort. B, Amino acid-level signal-to-noise analysis of radical variants found in *MYBPC3*.

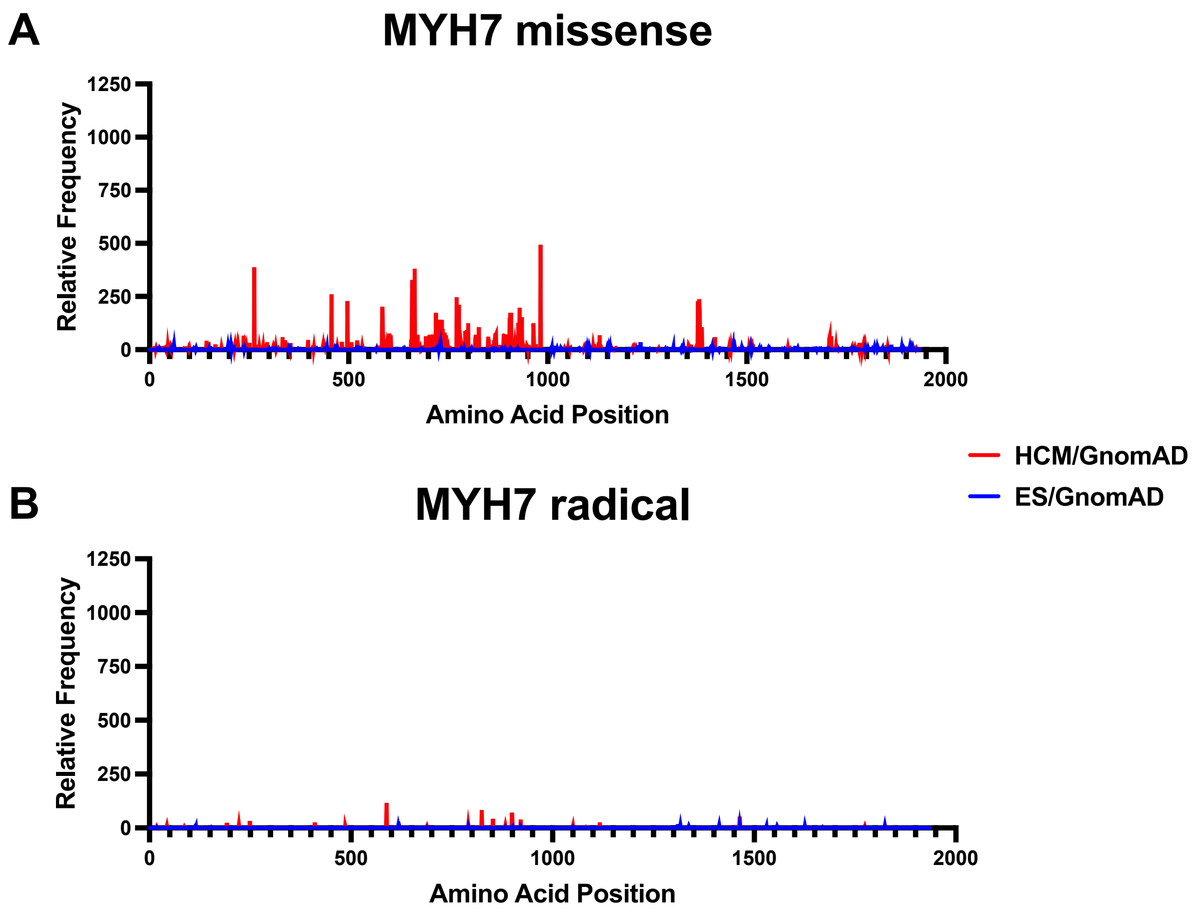

Supplemental Figure S3: A, Amino acid-level signal-to-noise analysis of missense variants found in *MYH7* for both HCM cases (red) and ES-identified variants (blue), compared with variants found in the gnomAD cohort. B, Amino acid-level signal-to-noise analysis of radical variants found in *MYH7*.

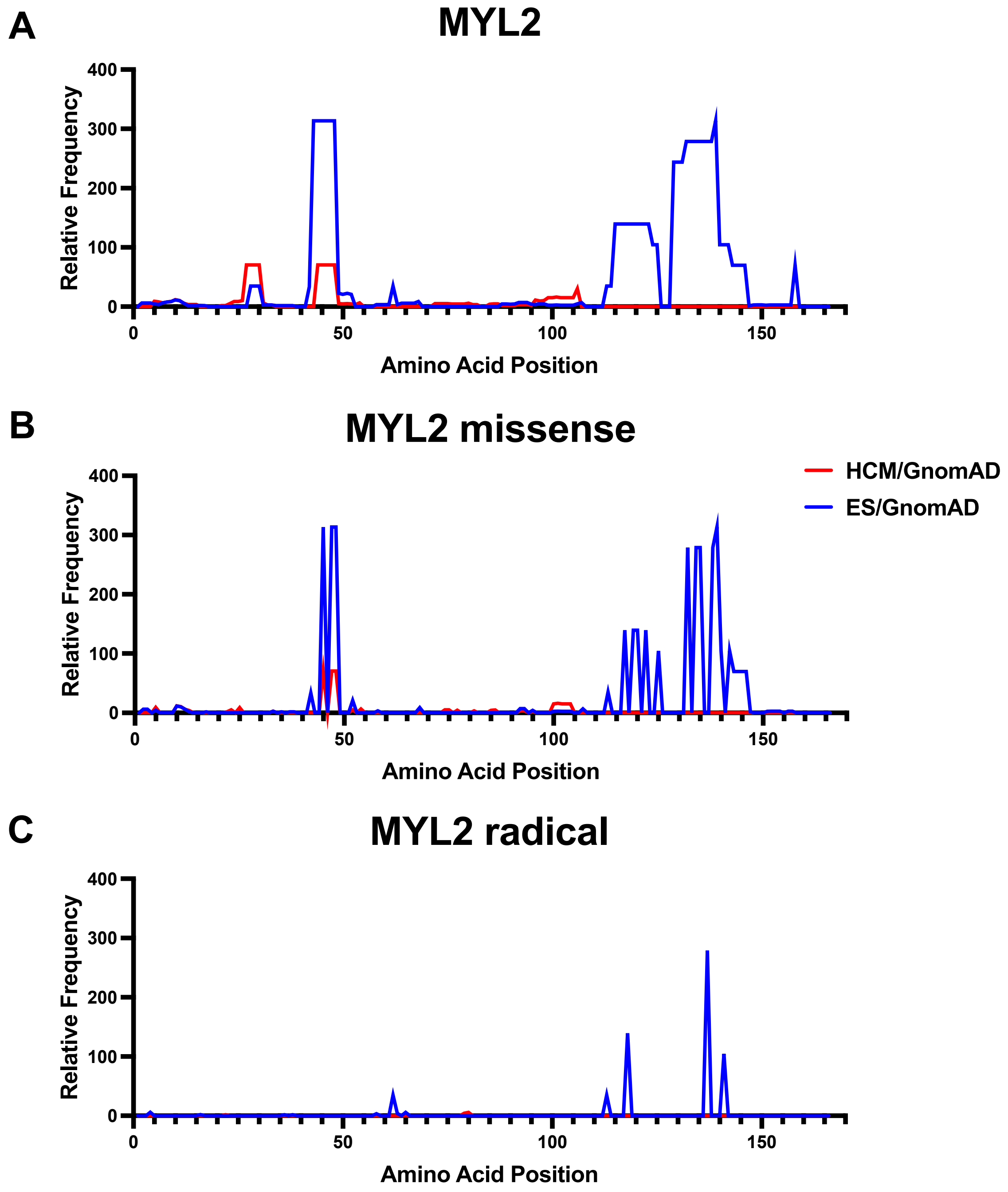

<u>Supplemental Figure S4:</u> A, Amino acid-level signal-to-noise analysis of all variants found in *MYL2* for both HCM cases (red) and ES-identified variants (blue), compared with variants found in the gnomAD cohort. B, Amino acid-level signal-to-noise analysis of missense variants found in *MYL2*. C, Amino acid-level signal-to-noise analysis of radical variants found in *MYL2*.

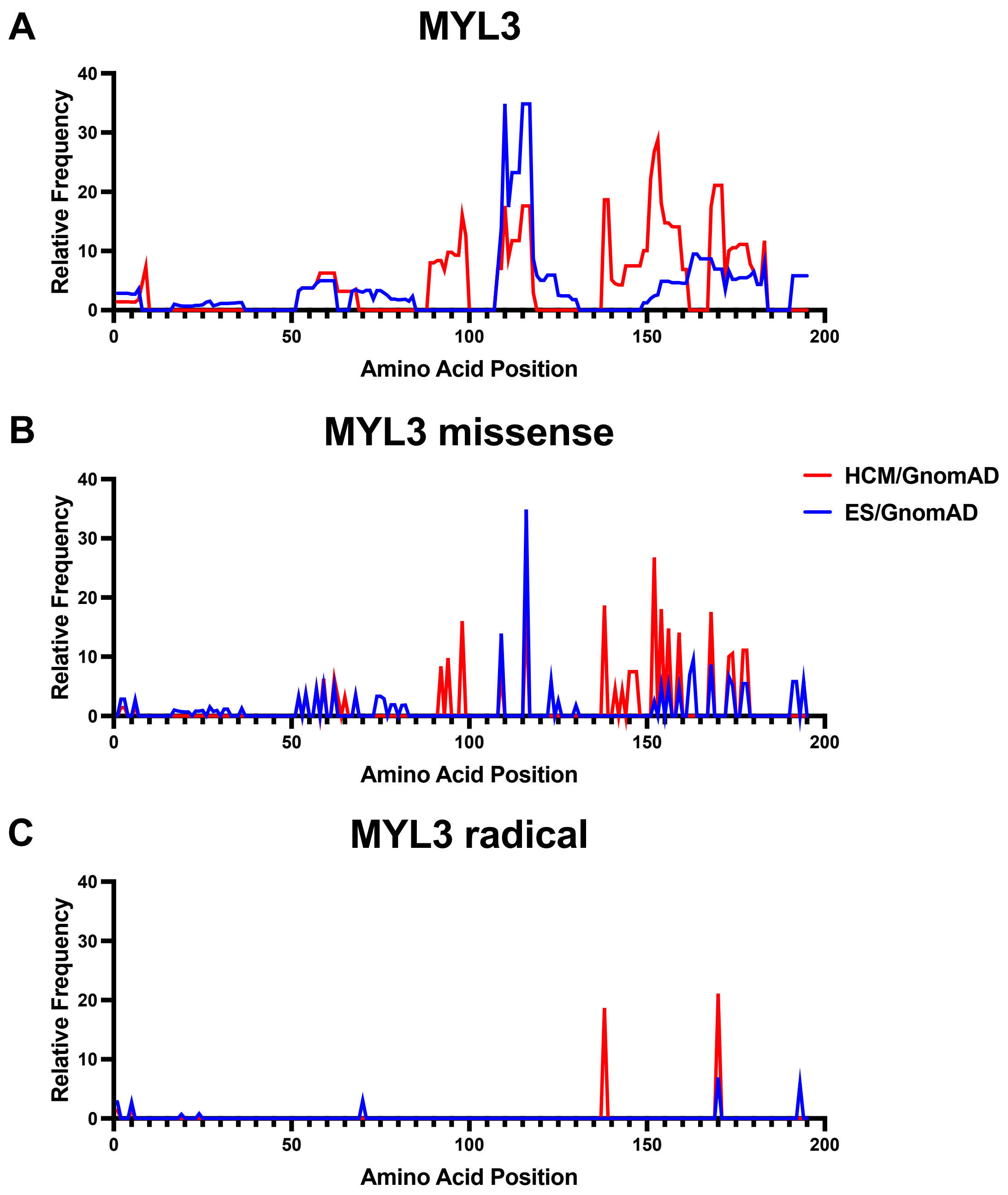

<u>Supplemental Figure S5:</u> A, Amino acid-level signal-to-noise analysis of all variants found in *MYL3* for both HCM cases (red) and ES-identified variants (blue), compared with variants found in the gnomAD cohort. B, Amino acid-level signal-to-noise analysis of missense variants found in *MYL3*. C, Amino acid-level signal-to-noise analysis of radical variants found in *MYL3*.

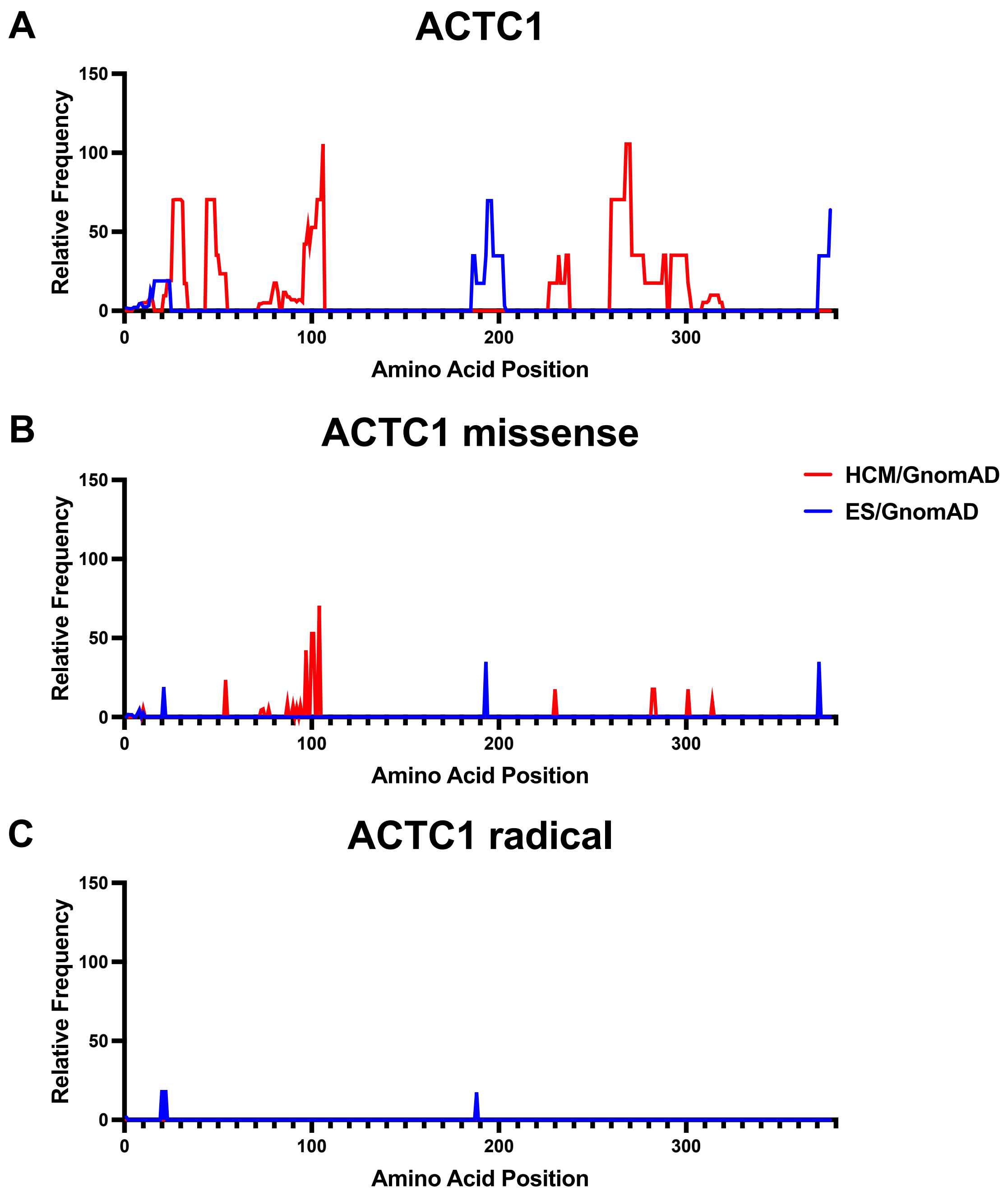

Supplemental Figure S6: A, Amino acid-level signal-to-noise analysis of all variants found in *ACTC1* for both HCM cases (red) and ES-identified variants (blue), compared with variants found in the gnomAD cohort. B, Amino acid-level signal-to-noise analysis of missense variants found in *ACTC1*. C, Amino acid-level signal-to-noise analysis of radical variants found in *ACTC1*.

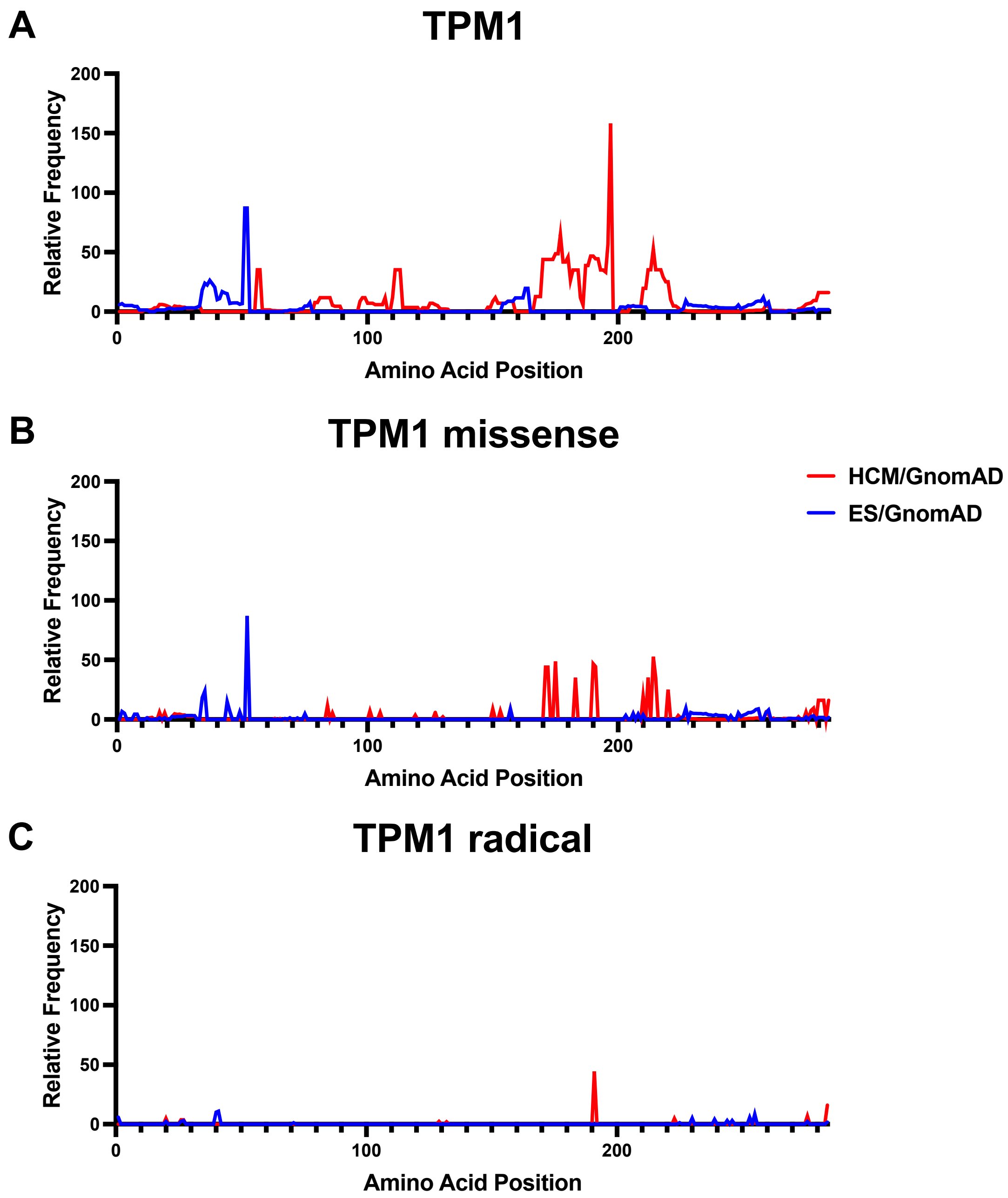

<u>Supplemental Figure S7:</u> A, Amino acid-level signal-to-noise analysis of all variants found in *TPM1* for both HCM cases (red) and ES-identified variants (blue), compared with variants found in the gnomAD cohort. B, Amino acid-level signal-to-noise analysis of missense variants found in *TPM1*. C, Amino acid-level signal-to-noise analysis of radical variants found in *TPM1*.

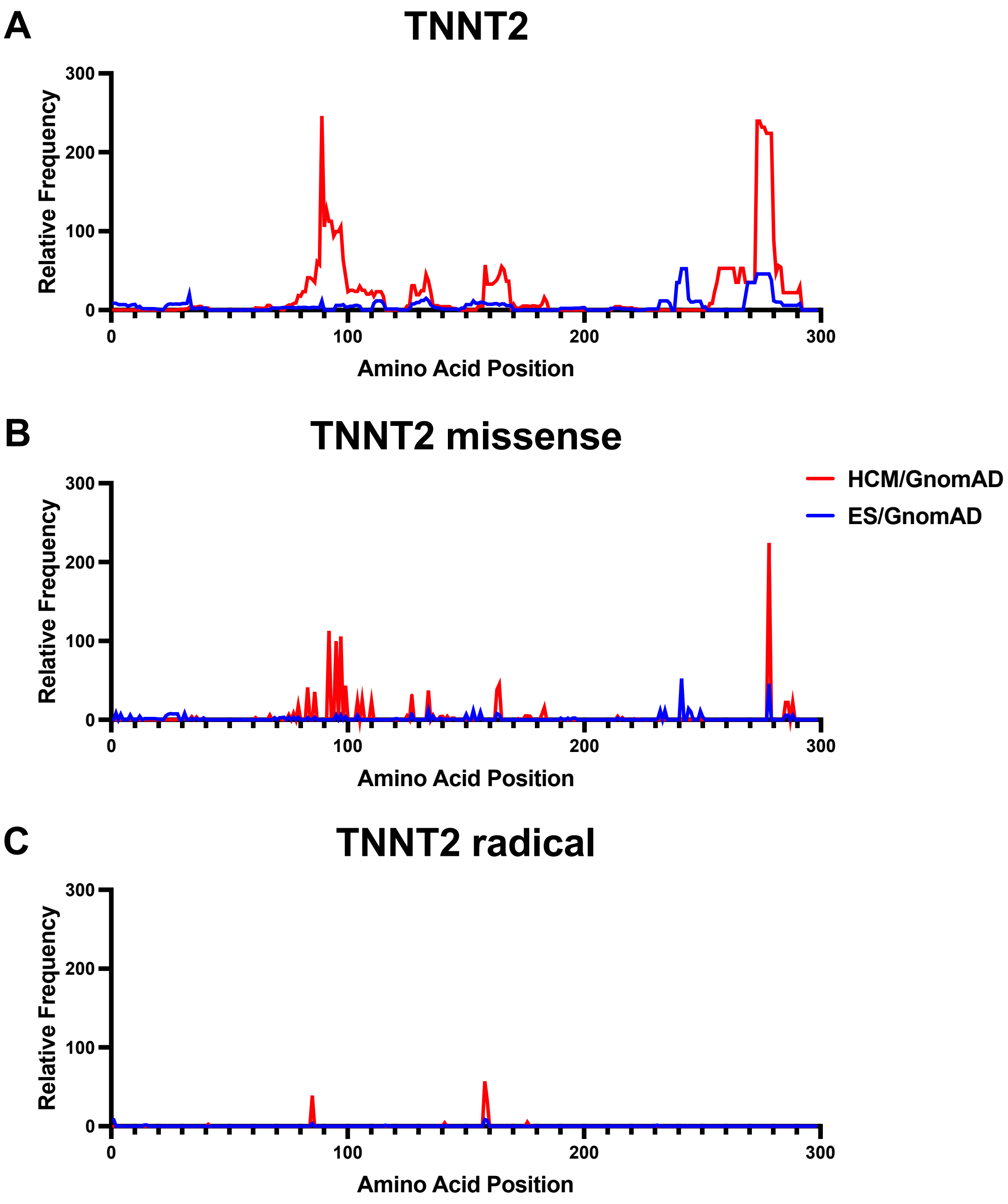

<u>Supplemental Figure S8:</u> A, Amino acid-level signal-to-noise analysis of all variants found in *TNNT2* for both HCM cases (red) and ES-identified variants (blue), compared with variants found in the gnomAD cohort. B, Amino acid-level signal-to-noise analysis of missense variants found in *TNNT2*. C, Amino acid-level signal-to-noise analysis of radical variants found in *TNNT2*.

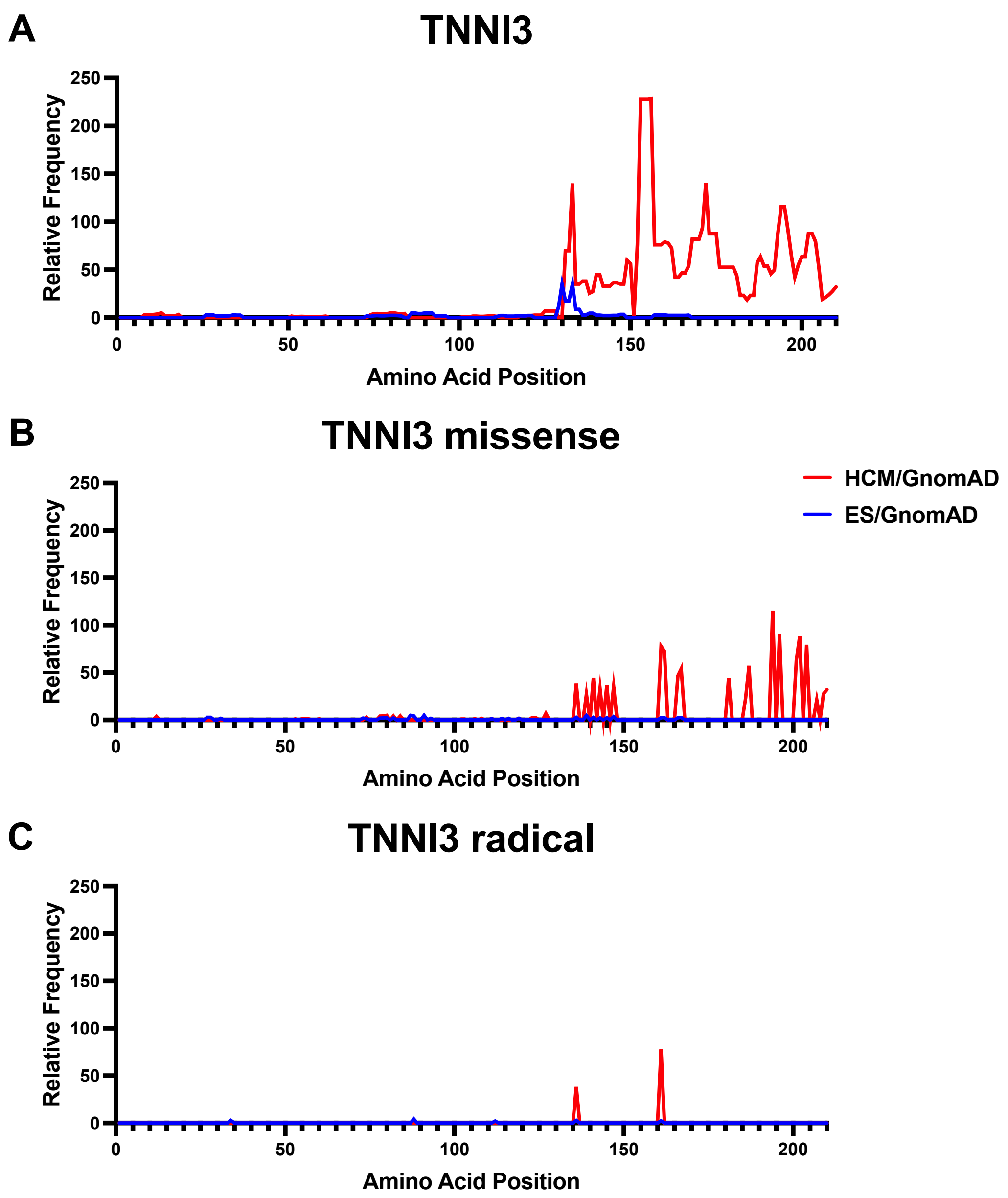

<u>Supplemental Figure S9:</u> A, Amino acid-level signal-to-noise analysis of all variants found in *TNNI3* for both HCM cases (red) and ES-identified variants (blue), compared with variants found in the gnomAD cohort. B, Amino acid-level signal-to-noise analysis of missense variants found in *TNNI3*. C, Amino acid-level signal-to-noise analysis of radical variants found in *TNNI3*.

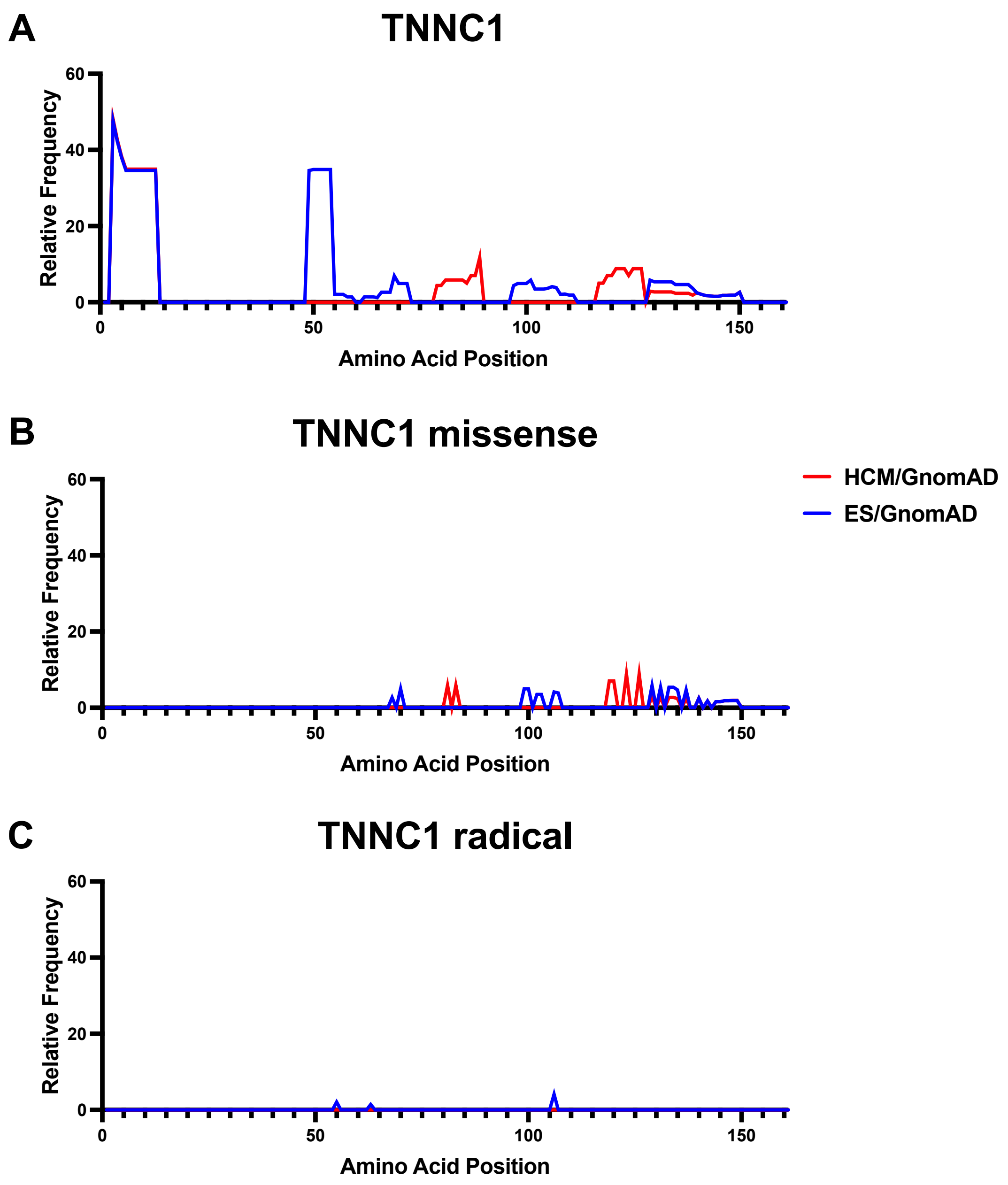

<u>Supplemental Figure S10:</u> A, Amino acid-level signal-to-noise analysis of all variants found in *TNNC1* for both HCM cases (red) and ES-identified variants (blue), compared with variants found in the gnomAD cohort. B, Amino acid-level signal-to-noise analysis of missense variants found in *TNNC1*. C, Amino acid-level signal-to-noise analysis of radical variants found in *TNNC1*.

SUPPLEMENTAL REFERENCES

1. Landstrom AP, Dailey-Schwartz AL, Rosenfeld JA, et al. Interpreting Incidentally Identified Variants in Genes Associated With Catecholaminergic Polymorphic Ventricular Tachycardia in a Large Cohort of Clinical Whole-Exome Genetic Test Referrals. *Circulation Arrhythmia and electrophysiology*. Apr 2017;10(4)doi:10.1161/CIRCEP.116.004742
2. Landrum MJ, Lee JM, Benson M, et al. ClinVar: improving access to variant interpretations and supporting evidence. *Nucleic Acids Res*. Jan 2018;46(D1):D1062-D1067. doi:10.1093/nar/gkx1153
3. Bos JM, Will ML, Gersh BJ, Kruisselbrink TM, Ommen SR, Ackerman MJ. Characterization of a phenotype-based genetic test prediction score for unrelated patients with hypertrophic cardiomyopathy. *Mayo Clin Proc*. Jun 2014;89(6):727-37. doi:10.1016/j.mayocp.2014.01.025
4. Alfares AA, Kelly MA, McDermott G, et al. Results of clinical genetic testing of 2,912 probands with hypertrophic cardiomyopathy: expanded panels offer limited additional sensitivity. *Genet Med*. Nov 2015;17(11):880-8. doi:10.1038/gim.2014.205
5. Richard P, Charron P, Carrier L, et al. Hypertrophic cardiomyopathy: distribution of disease genes, spectrum of mutations, and implications for a molecular diagnosis strategy. *Circulation*. May 6 2003;107(17):2227-32. doi:10.1161/01.CIR.0000066323.15244.54
6. Berge KE, Leren TP. Genetics of hypertrophic cardiomyopathy in Norway. *Clin Genet*. Oct 2014;86(4):355-60. doi:10.1111/cge.12286
7. Weissler-Snir A, Hindieh W, Gruner C, et al. Lack of Phenotypic Differences by Cardiovascular Magnetic Resonance Imaging in MYH7 (β-Myosin Heavy Chain)- Versus MYBPC3 (Myosin-Binding Protein C)-Related Hypertrophic Cardiomyopathy. *Circ Cardiovasc Imaging*. Feb 2017;10(2)doi:10.1161/CIRCIMAGING.116.005311
8. Morner S, Richard P, Kazzam E, et al. Identification of the genotypes causing hypertrophic cardiomyopathy in northern Sweden. *J Mol Cell Cardiol*. Jul 2003;35(7):841-9. doi:10.1016/s0022-2828(03)00146-9
9. Erdmann J, Daehmlow S, Wischke S, et al. Mutation spectrum in a large cohort of unrelated consecutive patients with hypertrophic cardiomyopathy. *Clin Genet*. Oct 2003;64(4):339-49. doi:10.1034/j.1399-0004.2003.00151.x
10. Olivotto I, Girolami F, Ackerman MJ, et al. Myofilament protein gene mutation screening and outcome of patients with hypertrophic cardiomyopathy. *Mayo Clin Proc*. Jun 2008;83(6):630-8. doi:10.4065/83.6.630
11. Andersen PS, Havndrup O, Hougs L, et al. Diagnostic yield, interpretation, and clinical utility of mutation screening of sarcomere encoding genes in Danish hypertrophic cardiomyopathy patients and relatives. *Hum Mutat*. Mar 2009;30(3):363-70. doi:10.1002/humu.20862
12. Rodriguez-Garcia MI, Monserrat L, Ortiz M, et al. Screening mutations in myosin binding protein C3 gene in a cohort of patients with Hypertrophic Cardiomyopathy. *BMC Med Genet*. Apr 30 2010;11:67. doi:10.1186/1471-2350-11-67
13. Millat G, Bouvagnet P, Chevalier P, et al. Prevalence and spectrum of mutations in a cohort of 192 unrelated patients with hypertrophic cardiomyopathy. *Eur J Med Genet*. Sep-Oct 2010;53(5):261-7. doi:10.1016/j.ejmg.2010.07.007
14. Waldmüller S, Erdmann J, Binner P, et al. Novel correlations between the genotype and the phenotype of hypertrophic and dilated cardiomyopathy: results from the German Competence Network Heart Failure. *Eur J Heart Fail*. Nov 2011;13(11):1185-92. doi:10.1093/eurjhf/hfr074
15. Brito D, Miltenberger-Miltenyi G, Vale Pereira S, Silva D, Diogo AN, Madeira H. Sarcomeric hypertrophic cardiomyopathy: genetic profile in a Portuguese population. *Rev Port Cardiol*. Sep 2012;31(9):577-87. doi:10.1016/j.repc.2011.12.020
16. Gruner C, Ivanov J, Care M, et al. Toronto hypertrophic cardiomyopathy genotype score for prediction of a positive genotype in hypertrophic cardiomyopathy. *Circ Cardiovasc Genet*. Feb 2013;6(1):19-26. doi:10.1161/CIRCGENETICS.112.963363

17. Zou Y, Wang J, Liu X, et al. Multiple gene mutations, not the type of mutation, are the modifier of left ventricle hypertrophy in patients with hypertrophic cardiomyopathy. *Mol Biol Rep*. Jun 2013;40(6):3969-76. doi:10.1007/s11033-012-2474-2
18. Lek M, Karczewski KJ, Minikel EV, et al. Analysis of protein-coding genetic variation in 60,706 humans. *Nature*. 08 2016;536(7616):285-91. doi:10.1038/nature19057
19. Whiffin N, Minikel E, Walsh R, et al. Using high-resolution variant frequencies to empower clinical genome interpretation. *Genet Med*. Oct 2017;19(10):1151-1158. doi:10.1038/gim.2017.26
20. Walsh R, Thomson KL, Ware JS, et al. Reassessment of Mendelian gene pathogenicity using 7,855 cardiomyopathy cases and 60,706 reference samples. Original Research Article. *Genet Med*. Feb 2017;19(2):192-203. doi:10.1038/gim.2016.90
21. Headrick AT, Rosenfeld JA, Yang Y, et al. Incidentally identified genetic variants in arrhythmogenic right ventricular cardiomyopathy-associated genes among children undergoing exome sequencing reflect healthy population variation. *Mol Genet Genomic Med*. Jun 2019;7(6):e593. doi:10.1002/mgg3.593
22. Hunt SE, McLaren W, Gil L, et al. Ensembl variation resources. *Database (Oxford)*. Jan 1 2018;2018doi:10.1093/database/bay119
23. Landstrom AP, Fernandez E, Rosenfeld JA, et al. Amino acid-level signal-to-noise analysis of incidentally identified variants in genes associated with long QT syndrome during pediatric whole exome sequencing reflects background genetic noise. *Heart Rhythm*. Jul 2018;15(7):1042-1050. doi:10.1016/j.hrthm.2018.02.031
24. Jones EG, Landstrom AP. Determining the Likelihood of Variant Pathogenicity Using Amino Acid-level Signal-to-Noise Analysis of Genetic Variation. *Journal of visualized experiments : JoVE*. Jan 16 2019;(143)doi:10.3791/58907
25. Tadros HJ, Life CS, Garcia G, et al. Meta-analysis of cardiomyopathy-associated variants in troponin genes identifies loci and intragenic hot spots that are associated with worse clinical outcomes. *J Mol Cell Cardiol*. May 2020;142:118-125. doi:10.1016/j.yjmcc.2020.04.005
26. Dean A, Sullivan K, Soe M. OpenEpi: Open Source Epidemiologic Statistics for Public Health, version 3.01. www.OpenEpi.com
27. Hitchcock-DeGregori SE, Song Y, Greenfield NJ. Functions of tropomyosin's periodic repeats. *Biochemistry*. Dec 2002;41(50):15036-44.
